# Formant frequencies and bandwidths of the vocal tract transfer function are affected by the mechanical impedance of the vocal tract wall

**DOI:** 10.1007/s10237-014-0632-2

**Published:** 2014-11-23

**Authors:** Mario Fleischer, Silke Pinkert, Willy Mattheus, Alexander Mainka, Dirk Mürbe

**Affiliations:** 1 Department of Otorhinolaryngology, Faculty of Medicine Carl Gustav Carus, Technische Universität Dresden, Dresden, Germany; 2 Division of Phoniatrics and Audiology, Department of Otorhinolaryngology, Faculty of Medicine Carl Gustav Carus, Technische Universität Dresden, Dresden, Germany

**Keywords:** Vocal tract transfer function, Acoustics, Finite element modelling, Wall impedance

## Abstract

The acoustical properties of the vocal tract, the air-filled cavity between the vocal folds and the mouth opening, are determined by its individual geometry, the physical properties of the air and of its boundaries. In this article, we address the necessity of complex impedance boundary conditions at the mouth opening and at the border of the acoustical domain inside the human vocal tract. Using finite element models based on MRI data for spoken and sung vowels /a/, /i/ and // and comparison of the transfer characteristics by analysis of acoustical data using an inverse filtering method, the global wall impedance showed a frequency-dependent behaviour and depends on the produced vowel and therefore on the individual vocal tract geometry. The values of the normalised inertial component (represented by the imaginary part of the impedance) ranged from $250\,\hbox {g}/\hbox {m}^{2}$ at frequencies higher than about 3 kHz up to about $2.5\times 10^{5}\,\hbox {g}/\hbox {m}^{2}$ in the mid-frequency range around 1.5–3 kHz. In contrast, the normalised dissipation (represented by the real part of the impedance) ranged from $65$ to $4.5\times 10^{5}\,\hbox {Ns}/\hbox {m}^{3}$. These results indicate that structures enclosing the vocal tract (e.g. oral and pharyngeal mucosa and muscle tissues), especially their mechanical properties, influence the transfer of the acoustical energy and the position and bandwidth of the formant frequencies. It implies that the timbre characteristics of vowel sounds are likely to be tuned by specific control of relaxation and strain of the surrounding structures of the vocal tract.

## Introduction

The human vocal tract (VT), the aeroacoustic cavity between the vocal folds and the open surface at the position of the lips, acts as a resonator of the pressure excitation due to the self-excited vocal folds motion and airflow modulation caused by and based on the lung pressure (Fant 1960). In contrast to the classical source-filter theory (Fant 1960), it has been shown that the VT is influencing the vocal fold vibration caused by its input impedance (Titze and Story 1997) and is partly responsible for characteristics of the source signal containing a fundamental frequency and higher integer harmonics which amplitudes decrease by about 12 dB/octave (see Doval et al. 2006; Mittal et al. 2013 for an overview). The topology of the frequency-dependent VT transfer function (TF) and its characteristic resonance frequencies, known as formant frequencies, is strongly determined by its geometry, especially the length and the area functions (Fant 1960; Story et al. 1998) and the junction impedance to the surrounding air, i.e. the near- and far-field acoustics (Vampola et al. 2011), and its interaction with the tissues inside the upper respiratory system (Sondhi 1974).

During speech and also during singing, all these mentioned features rapidly change at a split of a second (Sundberg et al. 1992). Therefore, the investigation of constantly sustained vowels is often used to describe the (quasi-static) physics of the VT (Takemoto et al. 2006; Clément et al. 2007; Echternach et al. 2011). Here, the position of the formant frequencies and the associated bandwidths determine the articulated vowel and its quality. Furthermore, the formant frequencies and the bandwidths depend on each other (Hawks and Miller 1995).

Up to now, it is not possible to measure the TF of the VT directly. Therefore, (semi-)automatic procedures, known as inverse filtering methods, are often used to assess the interesting voice source properties based on recorded audio or flow signals of the subject (Rothenberg 1973; Granqvist et al. 2003; Airas 2008; Lehto et al. 2007). In sum, the quality of these procedures has been recently confirmed to be satisfactory in a physical system (Chu et al. 2013).

To model the physics of the VT, several approaches are used. Considering that the sound wavelength is great compared with the cross-dimensions of the VT (Flanagan et al. 1975) and, therefore, assuming a one-dimensional wave propagation along the VT and under observing of adequate matching conditions, a stack of straightened uniform cylinders based on the VT area function can be used to solve the simplified wave equation (Sundberg et al. 1992; Boersma 1998).

This simplification of the governing equations mentioned above ignores the fact that the formant frequencies are also somewhat lowered because of the individual bent geometry of the VT (Sondhi 1986) and that the TF is affected by minor deviations from the cylinder-based shape due to lateral cavities such as the *sinus piriformes* (Vampola et al. 2013) and iatrogenic modifications (after tonsillectomy) (Švancara and Horáček 2006; Švancara et al. 2006).

In many cases, the determination of the area function is based on cross-sectional MRI data (Story et al. 1996; Baer et al. 1991) which can be directly analysed, without any assumption, using numerical methods, such as the finite element method (Švancara and Horáček 2006; Švancara et al. 2006; Vampola et al. 2008b; Motoki 2002) or the finite volume method in combination with the immersed boundary method (Mittal et al. 2011).

Apart from adequately defined material properties of the air, e.g. the speed of sound and density, all these modelling approaches require well-defined boundary conditions at the individual surface areas of the VT.

Looking at the acoustics, the entire surface of the VT wall can be divided in an area where the mouth opening is interacting with the adjacent air, and the VT wall, where the VT is in contact with surrounding soft and dense tissues, such as oral and pharyngeal mucosa, muscles and cartilages. At the mouth opening, the outgoing waves are partly absorbed which has been considered by means of different approaches: an additional end correction (Sundberg et al. 1992; Echternach et al. 2011), impedance boundary conditions to generate absorption of spherical waves (Sondhi 1974; Matsuzaki and Motoki 2011), impedance boundary conditions which is seen by an circular piston (Adachi and Yamada 1999; Vampola et al. 2008b) and an elliptic piston (Arnela and Guasch 2013) acting to an infinite baffle or by considering of the head geometry (Arnela et al. 2013). Furthermore, it has been suggested to model an hemispherical volume to the mouth region including adequate boundary conditions to force full absorption at the outer boundary (Motoki 2002). Regarding the VT wall, several conclusions were drawn. It was argued that wall impedance only acts at low frequencies (Sondhi 1974). Fujimura and Lindqvist (1971) found that the great bandwidth of the first formant is caused by non-rigid walls. In the low-frequency range up to 150 Hz, measurements of the wall impedance show a complex mechanical behaviour (Ishizaka et al. 1975). It can be modelled as a radiation impedance as found for a pulsating cylinder up to 500 Hz (Flanagan et al. 1975). Fant et al. (1976) suggested a non-uniform distribution of mass along the VT. In a study, the upper limit of the investigated frequency range was of a several hundred Hz (Fant et al. 1976). In contrast, in finite element models, only the real part of the impedance—which correlates to energy losses— has been introduced by formulating a specific impedance as assumed for soft tissue material (Švancara et al. 2006; Vampola et al. 2008a, b). Furthermore, a frequency-dependent complex wall impedance based on a 2 cm thick soft cylindrical wall has been used to calculate the TF up to a frequency of 10 kHz Matsuzaki and Motoki (2007, 2011).

Nevertheless, several questions regarding the complex physics of the TF remain and will be addressed in this article: how can the domain outside the VT be considered in a simple but accurate way? How is the (uniformly) distributed complex wall impedance influencing both, the formant frequencies and the bandwidths, under consideration of geometrically realistic VT models? Are there any differences in the impedances during articulation of different vowels? Does the wall impedance change if a subject switches from speech to singing mode?

## Materials and methods

### Data aquisition and model creation

For this study, we analysed the VT geometry by using magnetic resonance tomography (MRI; MAGNETOM Trio, A Tim System 3T, Siemens Medical Solutions, Erlangen, Germany) of one male subject (22 years old; baritone; classical singing student from the Hochschule für Musik Carl Maria von Weber, Dresden, Germany). The subject was instructed for sustained voice production of the vowels /a/, /i/ and // for up to 9.2 s, both in normal speech voice production and in singing voice production as being typically used in classical operatic singing. Due to superimposition of the noise within the MRI, the audio signal was recorded immediately (15–30 min) after acquisition of the image data. The subject repeated the task outside the MRI machine in a semi-anechoic chamber in the same recumbent position, and the acoustical output was recorded via a condenser microphone (t.bone EM-900, Thomann, Burgebrach, Germany). Throughout the measurements, the subject was instructed to keep the fundamental frequency constant at 220 Hz. This was supported by the available signal of a pitch pipe and was checked before and after the recordings by means of fundamental frequency analysis.

The acoustical data were then analysed by using the inverse filtering software DECAP (Svante Granqvist, Department of Speech, Music and Hearing, KTH, Sweden) for estimating the formant frequencies and the bandwidths. Detailed information on the inverse filtering method and the usage of DECAP are given in Sundberg et al. (2013).

**Fig. 1:**
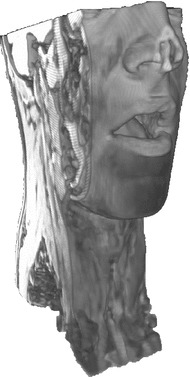
Example of a three-dimensional stack based on MRI recordings, where the subject produces the vowel /a/ in singing mode

**Fig. 2:**
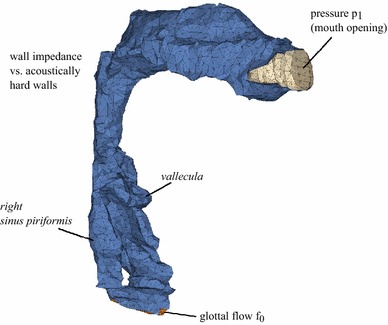
Finite element model of the VT for the vowel /a/ in singing mode. Labels and *different colours* denote three distinctive surface areas where specific boundary conditions were applied. Among the main cavity of the VT, the *vallecula* and the *sinus piriformes* were incorporated into the model

The images, which were scanned in the sagittal plane, were automatically combined to a voxel-based three-dimensional stack (Fig. 1). Based on these image stacks, we segmented the VT cavity using IPTOOLS, a segmentation software which has been successfully used in segmentation of tubular cavities as described in Poznyakovskiy et al. (2008) and Poznyakovskiy et al. (2011). In additional to the main cavity between the vocal fold plane and the lips region, the high resolution allowed the inclusion of small cavities such as the *vallecula* and the *sinus piriformes* (see Fig. 2). As already known, because of the property of the MRI to visualise differences of the water content inside the structures, we were not able to detect the teeth (because of missing water inside). But here, for the chosen vowels and configurations, disregarding the teeth seems to be negligible. In case of the vowel /i/, the tongue is reducing the oral cavity to a small palatal passage, and in case of /a/ and //, teeth may have a limited significance, since the cross-sectional area in the oral region is large. The segmented surface was then exported to the freely available software GMSH (http://geuz.org/gmsh/), and a tetrahedral volume mesh was created and reprocessed within the finite element solver ANSYS, V14 (ANSYS, Inc., Canonsburg, PA). Here, for calculation, we used the element type FLUID221 that exhibits quadratic pressure behaviour. Furthermore, the number of elements of the mesh was chosen to ensure the quality of the results up to 4 kHz.

### Numerical modelling

Assuming a compressible fluid without any mean flow, a constant density $\rho _0$ of $1.15\,\hbox {kg}/\hbox {m}^{3}$ and—because of short distances of wave propagation—a negligible bulk viscosity, and a harmonic time dependence of the sound pressure $p(\mathbf{x},t)=\mathrm{Re}\lbrace \tilde{p}(\mathbf{x})\,e^{\text{ j }\omega t} \rbrace$ the governing Helmholtz equation reads as follows:

1 $$\begin{aligned} \displaystyle \left( -\kappa ^2-\nabla ^2\right) \tilde{p}=0\quad \text{ in } \text{ V. } \end{aligned}$$

Herein, $\nabla$ is the nabla operator, $\kappa =\omega /c$ is the wave number, $\omega$ is the angular frequency and $c$ is the constant speed of sound which was set to 350 m/s in all subsequent computations.

As explained in detail below, we divided the surface of the models into three distinctive areas: the glottal area, the curved surface of the mouth opening and the surface of the VT at the air–tissue border (Fig. 2).

Therefore, we included the following boundary conditions,

2 $$\begin{aligned} \nabla \tilde{p}\cdot \mathbf{n}&= \tilde{f}_0\quad \text{ on } \varGamma _\text {glottis}\nonumber \\ \displaystyle \nabla \tilde{p}\cdot \mathbf{n}&= -\text{ j }\kappa \frac{c\rho _0}{Z_\text {mouth}}\tilde{p}\quad \text{ on }\,\varGamma _\text {mouth}\nonumber \\ \displaystyle \nabla \tilde{p}\cdot \mathbf{n}&= -\text{ j }\kappa \frac{c\rho _0}{Z_\text {wall}}\tilde{p}\quad \text{ on } \varGamma _\text {wall} \end{aligned}$$

where $\mathbf{n}$ is the outward normal vector. Considering the weak form by multiplying by the test function $q$, integrating over the volume $V$ of the computational domain and applying the Gauss Theorem, Eq. 1 becomes

3 $$\begin{aligned}&\displaystyle -\kappa ^2\int _{V}q\,\tilde{p}\,\mathrm{d}V+\int _{V}\nabla q\cdot \nabla \tilde{p}\,\mathrm{d}V\nonumber \\&\quad \displaystyle =-\!\int _{\varGamma _\text {mouth}}\text{ j }\frac{\kappa c\rho _0}{Z_\text {mouth}}\,q\,\tilde{p}\;\mathrm{d}\varGamma _\text {mouth} \!-\!\int _{\varGamma _\text {wall}}\text{ j }\frac{\kappa c\rho _0}{Z_\text {wall}}\,q\,\tilde{p}\;\mathrm{d}\varGamma _\text {wall}\nonumber \\&\qquad +\displaystyle {\int _{\varGamma _\text {glottis}}q\,\tilde{f}_0\;\mathrm{d}\varGamma _\text {glottis}}. \end{aligned}$$

Here, $Z_\text {mouth}$ and $Z_\text {wall}$ are the complex impedances applied at the corresponding surfaces of the acoustic domain. It can be imagined as an impedance sheet which couples the pressure with the acoustic velocity vector normal to the boundary.

If we attempt a finite element solution to the variational form in Eq. 3 and, respectively, approximate the acoustic pressure and test function by $\tilde{p}\simeq \tilde{p}_h=\mathbf{N}^{T}\mathbf{P}$ and $\tilde{q}\simeq \tilde{q}_h=\mathbf{N}^{T}\mathbf{P}$ ($\mathbf{N}$ being a vector of basis functions and $\mathbf{P}$ and $\mathbf{Q}$ the unknown vector of nodal pressure values and nodal test function values), we get, after substitution in Eq. 3, the following matrix system:

4 $$\begin{aligned} \displaystyle \left( -\omega ^2\mathbf{M}+\text {j}\omega \,\mathbf{D}+\mathbf{K}\right) \mathbf{P}=\mathbf{0}. \end{aligned}$$

Here, $\mathbf{M}$, $\mathbf{D}$ and $\mathbf{K}$ are the mass, damping, and stiffness matrices, respectively. According to Eq. 3, the damping matrix $\mathbf{D}$ becomes

5 $$\begin{aligned} \displaystyle \mathbf{D}&= \frac{\rho _0\,\mathrm{Re}(Z_\text {mouth})}{\mathrm{Re}(Z_\text {mouth})^2+ \mathrm{Im}(Z_\text {mouth})^2} \int _{(\varGamma _\text {mouth})}\mathbf{N}\mathbf{N}^{T}\;\mathrm{d}\varGamma _\text {mouth}\nonumber \\&+\,\displaystyle \frac{\rho _0\,\mathrm{Re}(Z_\text {wall})}{\mathrm{Re}(Z_\text {wall})^2+\mathrm{Im}(Z_\text {wall})^2} \int _{(\varGamma _\text {wall})}\mathbf{N}\mathbf{N}^{T}\;\mathrm{d}\varGamma _\text {wall} \end{aligned}$$

and the stiffness matrix $\mathbf{K}$ becomes

6 $$\begin{aligned} \displaystyle \mathbf{K}\!&= \!\mathbf{K}_0\nonumber \\&+\,\displaystyle \omega \frac{\rho _0\,\mathrm{Im}(Z_\text {mouth})}{\mathrm{Re}(Z_\text {mouth})^2\!+\!\mathrm{Im}(Z_\text {mouth})^2} \int _{(\varGamma _\text {mouth})}\mathbf{N}\mathbf{N}^{T}\;\mathrm{d}\varGamma _\text {mouth}\nonumber \\&+\,\displaystyle \omega \frac{\rho _0\,\mathrm{Im}(Z_\text {wall})}{\mathrm{Re}(Z_\text {wall})^2\!+\!\mathrm{Im}(Z_\text {wall})^2} \int _{(\varGamma _\text {wall})}\mathbf{N}\mathbf{N}^{T}\;\mathrm{d}\varGamma _\text {wall} \end{aligned}$$

where $\mathbf{K}_0$ denotes the stiffness of the undamped system. It is obvious that because of $Z$, both $\mathbf{D}$ and $\mathbf{K}$ are frequency dependent. It is further obvious that in case of $\mathrm{Re}(Z)\rightarrow 0,\, \mathbf{D}$ becomes $\mathbf{0}$, and in case of $\mathrm{Im}(Z)\rightarrow 0,\, \mathbf{K}$ becomes $\mathbf{K}_0$. Interestingly, if the real part and/or the imaginary part of the impedances tends to infinity, then, $\mathbf{D}$ becomes $\mathbf{0}$ and $\mathbf{K}$ becomes $\mathbf{K}_0$. That means, the systems tends to have acoustically hard walls. It should be noted that introducing an imaginary part of $Z$ can also be interpreted as a change of the mass matrix $\mathbf{M}$.

At the mouth region, for simplification, we coupled the degree of freedom resulting in a set of nodes. Thus, the pressure calculated for one node becomes the same for all nodes of the region. Further, we investigated the impact of three different impedances $Z_0$ on the TF. Firstly, we set

7 $$\begin{aligned} Z_0^\text {S}=\rho \displaystyle _0\,c\left( \frac{(\kappa \,r)^2}{1+ (\kappa \,r)^2}+\text {j}\,\frac{\kappa \,r}{1+(\kappa \,r)^2}\right) \end{aligned}$$

with $r=\sqrt{A_\text {mouth}/2\pi }$ (see Table 2), corresponding to a radiating half-sphere with a cross-sectional area equal to the lip opening to cause the absorption of spherical waves as suggested by Sondhi (1974). Secondly, we set $Z_0^\text {P}$ equal to the impedance of a rigid circular piston with radius $a=\sqrt{A_\text {mouth}/\pi }$ (see Table 2) that acts into an infinite baffle according to Morse and Ingard (1968). This model assumes the propagation of plane waves in a duct that impinge on an aperture. However, due to the even non-plane opening at the mouth, this model might not match the impedance exactly. Yet, this approach has been suggested in former research (Vampola et al. 2008b). Thirdly, we used the low-frequency approximation of

8 $$\begin{aligned} Z_0^\text {P}\approx Z_0^\text {LP}=\rho \displaystyle _0\,c\left( \frac{1}{2}\left( {\kappa \,a}\right) ^2\displaystyle +\text {j}\frac{8}{3\,\pi }{\kappa \,a}\right) \end{aligned}$$

according to Boersma (1998).

At the surface which is in contact with soft tissues, for simplification, we used an impedance formulation, containing a scalar mass $m$ coupled with a damper $b$, e.g. $Z_\text {wall}=\frac{b}{A}+\text {j} \omega \frac{m}{A}$ and normalised to the area $A$ of the wall. It should be noted that $Z_\text {wall}$ can be considered as an array of local mass–damper systems at all finite elements at the surface normal to the wall, and therefore, no exact fluid–structure interaction with the surrounding tissues was modelled. Because $m/A$ and $b/A$ are generally unknown, we did an extensive parameter study to examine their influence on the TF, the formant frequencies and their bandwidths. According to the analysis used in DECAP (Sundberg et al. 2013), the formants were computed based on the TF of the ratio of the (simplified) flow at the mouth to the glottal flow

9 $$\begin{aligned} \displaystyle TF(\omega )=\left| \tilde{p}_1(\omega )/(\text {j}\omega \cdot \tilde{f}_0(\omega ))\right| , \end{aligned}$$

where $\tilde{p}_1$ is the pressure at the mouth opening.

We sampled $m/A$ at $[{\bar{m}}_1,\bar{m}_2,\ldots ,\bar{m}_k,{\bar{m}}_{k+1},\ldots ,{\bar{m}}_l]/A$ and $b/A$ at $[\bar{b}_1,\bar{b}_2,\ldots ,\bar{b}_n,\bar{b}_{n+1},\ldots ,\bar{b}_o]/A$ in order to calculate the formant frequencies $\hbox {F}_{k,n}$ and bandwidths $\hbox {BW}_{k,n}$ piecewise at a rectangular grid (see Fig. 5 for details). Then, we used local approximation functions that read

10 $$\begin{aligned}&\text {F}(m/A,b/A)=[m/A,b/A,m\,b/A^{2},1]\,\mathbf{\psi }\nonumber \\&\text {BW}(m/A,b/A)=[m/A,b/A,m\,b/A^{2},1]\,\mathbf{\xi }. \end{aligned}$$

These functions describe the analytical dependence of F and BW each within the range of ${\bar{m}}_k/A\le m/A\le {\bar{m}}_{k+1}/A$ and $\bar{b}_n/A\le b/A\le \bar{b}_{n+1}/A$. The unknown vectors ${\psi }$ and ${\xi }$ were calculated by solving the linear matrix systems

11 $$\begin{aligned}&{\psi }\!=\!\left[ \begin{array}{llll} {\bar{m}}_{k}/A &{} {\bar{b}}_{n}/A &{} {\bar{m}}_{k}{\bar{b}}_{n}/A^{2} &{} 1\\ {\bar{m}}_{k+1}/A &{} {\bar{b}}_{n}/A &{} {\bar{m}}_{k+1}{\bar{b}}_{n}/A^{2} &{} 1\\ {\bar{m}}_{k}/A &{} {\bar{b}}_{n+1}/A &{} {\bar{m}}_{k}{\bar{b}}_{n+1}/A^{2} &{} 1\\ {\bar{m}}_{k+1}/A &{} {\bar{b}}_{n+1}/A &{} {\bar{m}}_{k+1}{\bar{b}}_{n+1}/A^{2} &{} 1\\ \end{array}\right] ^{-1}\left[ \begin{array}{l} \text {F}_{k,n}\\ \text {F}_{{k+1},n}\\ \text {F}_{{k},{n+1}}\\ \text {F}_{{k+1},{n+1}} \end{array}\right] \nonumber \\&{\xi }\!=\!\left[ \begin{array}{llll} {\bar{m}}_{k}/A &{} {\bar{b}}_{n}/A &{} {\bar{m}}_{k}{\bar{b}}_{n}/A^{2} &{} 1\\ {\bar{m}}_{k+1}/A &{} {\bar{b}}_{n}/A &{} {\bar{m}}_{k+1}{\bar{b}}_{n}/A^{2} &{} 1\\ {\bar{m}}_{k}/A &{} {\bar{b}}_{n+1}/A &{} {\bar{m}}_{k}{\bar{b}}_{n+1}/A^{2} &{} 1\\ {\bar{m}}_{k+1}/A &{} {\bar{b}}_{n+1}/A &{} {\bar{m}}_{k+1}{\bar{b}}_{n+1}/A^{2} &{} 1\\ \end{array}\right] ^{-1} \left[ \begin{array}{l} \text {BW}_{k,n}\\ \text {BW}_{{k+1},n}\\ \text {BW}_{{k},{n+1}}\\ \text {BW}_{{k+1},{n+1}} \end{array}\right] .\nonumber \\ \end{aligned}$$

This procedure was applied in order to get multiple objective functions that were analysed individually in order to find either the optimal formant frequencies or bandwidths (represented as a level curve) as determined with DECAP. The optimal combination of $m/A$ and $b/A$ to match the formant frequency and the bandwidth is represented as the intersection of two associated level curves. For this reason, we analysed the individual quadratic functions for $m/A$ and $b/A$ in Eq. 10 in order to match F and BW as determined with DECAP. Therefore, zeros of these quadratic functions were calculated analytically.

In a preliminary study, we estimated the lower limits of $b/A$ and $m/A$ to $1\,\hbox {N}\,\hbox {s}/\hbox {m}^3$ and $2.5\times 10^2\,\hbox {g}/\hbox {m}^2$, respectively. In those cases, the formant frequencies were shifted to values outside the interesting frequency range below 4 kHz. The upper limits for these parameters were estimated to be $10^5\,\hbox {N}\,\hbox {s}/\hbox {m}^3$ for $b/A$ and $2.5\times 10^5\,\hbox {g}/\hbox {m}^2$ for $m/A$, where the VT walls tend to have a very high impedance which is identical to acoustically hard walls, and therefore equivalent to a homogeneous Neumann boundary condition, e.g. $\nabla \tilde{p}\cdot \mathbf{n}=0$. In the present study, we only analysed TFs where the formants can clearly be separated and identified. We always solved Eq. 4 by doing a harmonic analysis in the frequency domain. In the computations, the frequency resolution was always 5 Hz. To solve the linear matrix system in the frequency domain as formulated in Eq. 4, the direct sparse solver as provided by ANSYS was used. Therefore, we expect numerical errors to be very low compared with the solved quantities of $\mathbf{P}$. All computations presented in this article were done on a normal desktop computer (Intel Core$^{\text {TM}}$ i5-2500, CPU 4 $\times$ 3.30GHz with 24 Gbyte memory) and took at least 1,680 h calculation time for all models and configurations.

## Results

### Acoustical properties

As an initial step of our work, we determined the relevant acoustical data of the VT, which means the formant frequencies and the bandwidths. We focussed on the first five formants which are of great interest for the perception of vowel quality and timbre. The results of the analysis using an inverse filtering method are shown in Table 1. It is obvious that the first three formants and bandwidths for the vowel /a/ are only slightly affected by the mode of voice production ($\le$9 Hz in formant frequencies and $\le$19 Hz in bandwidths), which means that there is no great difference between the singing or speech mode. The differences in the acoustical characteristics are greater for the fourth and fifth formant ($>$150 Hz) which is caused by the higher sensitivity to small geometric variations and/or physical properties of the VT by diminishing the wavelength. The results for the vowel /i/ are similar, but in contrast to the vowel /a/, and there is already an distinguished difference in the second formant in the order of $\approx$100 Hz observable, but the first and the third formant frequency (and their associated bandwidth) are nearly unaffected by the mode of voice production. Similar to vowel /a/, the fourth and the fifth formant are shifted in the singing mode relative to their position in case of the spoken vowel. For vowel //, the first three formant frequencies were slightly greater in singing mode, whereas the associated bandwidths were only slightly affected. A substantial difference of more than 500 Hz between the singing and speech mode was found at F5. This indicates that in the speech mode at about 3.5 kHz, a resonance was suppressed rather than a shift of a formant occurred. Summarising these (intermediate) data, in comparison with an overview given in Hawks and Miller (1995) and graphically shown in Fig. 8 (denoted with diamonds), the relationship between the formant frequencies and the bandwidth is in a plausible order of magnitude.

It should be noted that the inverse filtering procedure we used might have its own limitations caused by the semi-empirical components in its usage. That means, the acoustical values derived from DECAP, we defined as reference values, might be somewhat biased.

**Table 1:** Determined formant frequencies (F) in kHz and bandwidths (BW) in Hz for the individual vowels as denoted in the subheadings of the tables after the inverse filtering of the audio signal

	Singing mode		Speech mode	
No.	F	BW	F	BW
/a/				
1	0.555	68	0.564	49
2	1.120	33	1.120	44
3	2.468	69	2.468	69
4	2.737	74	3.089	79
5	3.301	140	3.473	142
/i/				
1	0.360	49	0.312	33
2	1.700	38	1.796	54
3	2.313	59	2.292	82
4	2.827	74	2.925	100
5	3.751	135	3.348	113
//				
1	0.409	38	0.379	36
2	1.356	42	1.218	42
3	2.533	67	2.469	79
4	2.819	79	2.928	82
5	3.301	140	3.873	186

### Geometrical properties

**Fig. 3:**
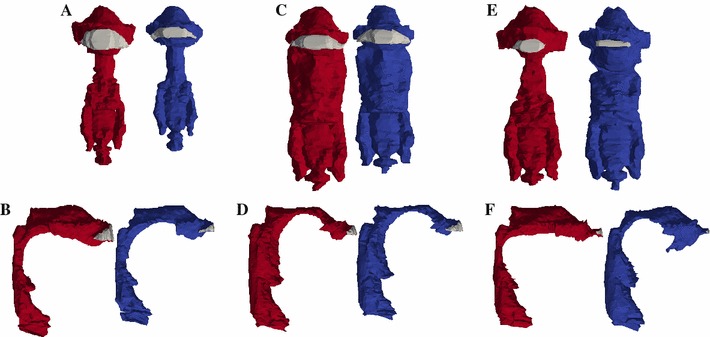
Finite element models of the VT in singing (*red coloured*) and speech mode (*blue coloured*) in the frontal view (**a**, **c**, **e**) and the sagittal view (**b** ,**d**, **f**), respectively, for the vowel /a/ (**a**, **b**), /i/ (**c**, **d**) and // (**e**, **f**). Besides the main cavity, the models include the *sinus piriformes* and the *vallecula*

As mentioned in the method section (Sect. 2), four different models (vowels /a/, /i/ and //, both in speech and singing mode) were examined. In general, as expected, distinctive differences between the vowels and the mode of voice production can be seen (Fig. 3). An overview on important geometrical measures is given in Table 2. It can be seen that not only the length of the VT depends on the mode of voice production (about 6.6–11 % longer in the singing mode), but also the size of the surface areas connected to the surrounding air (mouth opening) and the size of the area which is in contact with the soft tissues surrounding the VT. It should be noted that in case of the vowel /a/, the size of the mouth opening area in the singing mode is increased by the factor 2.3 in comparison with the speech mode of the same vowel. Further, the ratio of the surface area which connects to the tissue and the area of the mouth opening is between 24.4 and 179.3 depending on the vowel and mode of voice production.

**Table 2:** Details of geometrical measures used for calculation of various impedance values at the mouth opening and the internal fluid–structure interface within the VT

Vowel	Singing mode			Speech mode		
	Surface area $A_\text {mouth}$ of the mouth in $\hbox {mm}^{2}$	Surface area of the internal surface in $\hbox {mm}^{2}$	length of the VT at the mean path in cm	Surface area $A_\text {mouth}$ of the mouth in $\hbox {mm}^{2}$	Surface area of the internal surface in $\hbox {mm}^{2}$	Length of the VT at the mean path in cm
/a/	830.8	20,287	18.81	360.4	16,261	16.96
/i/	317.0	18,103	17.35	380.5	16,096	16.28
//	214.4	19,035	18.30	115.2	20,656	18.43

**Fig. 4:**
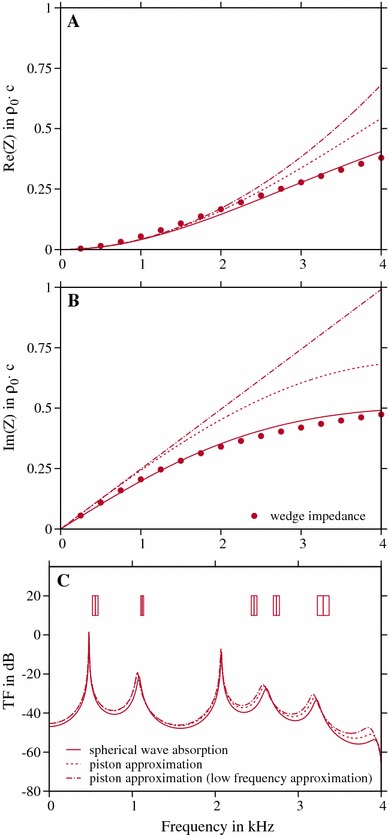
Comparison of different impedances as applied at the mouth opening for the vowel /a/ in the singing mode where the mouth is widely opened (**a**, **b**). Despite great variation between the different approaches as denoted in the legend, the resulting TFs are only slightly affected by the impedance as applied at mouth surface (**c**). For comparison, the middle vertical *lines* in the *boxes* in the upper part of **c** indicate the formant frequencies as determined with DECAP, and the width of the box is equivalent to the individual bandwidth, respectively

### Influence of the impedance at the mouth opening

As mentioned above and in Sect. 2, the mouth opening produces sounds like a monopole (Seo and Mittal 2011) which should be adequately approximated by formulation of an impedance $Z_0^\text {S}$. Considering Table 2, the greatest opening surface was found for vowel /a/ in the singing mode. Here, a deviation of the monopole approximation, especially at higher frequencies, could occur. In combination with acoustically hard VT walls and the real and the imaginary part of $Z_0^\text {S}$ as shown in Fig. 4a, b, the resulting TF is shown in Fig. 4c. In comparison with the formant frequencies and bandwidth as determined with DECAP (see boxes in Fig. 4c), a great discrepancy can be seen in the whole frequency range (see also the results for all models, denoted with circles, considering acoustically hard VT walls in Fig. 8). Therefore, we calculated the TF with two alternative approaches, $Z_0^\text {P}$ and $Z_0^\text {LP}$ according to Sect. 2 in order to see whether there is an enhancement of the model in order to get a better agreement with the values determined with DECAP. As shown in Fig. 4), despite the great variance in the impedance values, no significant enhancement of the model can be observed. Only small shifts of the peaks at higher frequencies occur by changing the impedance at the mouth. Considering the wedge-like shape of the mouth opening (see Fig. 3), we calculated the impedance of a wedge acting into an infinite baffle by using a simple finite element model (Fleischer et al. 2013) and plotted the resulting real and imaginary part with dots in Fig. 4a, b. The good agreement with $Z^\mathrm{S}_0$ shows that assuming perfect absorption of spherical waves into the outer space is an adequate modelling approach. Therefore $Z^\mathrm{S}_0$ is used in the subsequent analysis of the other models.

### Determination of the mechanical impedance of the VT wall

**Fig. 5:**
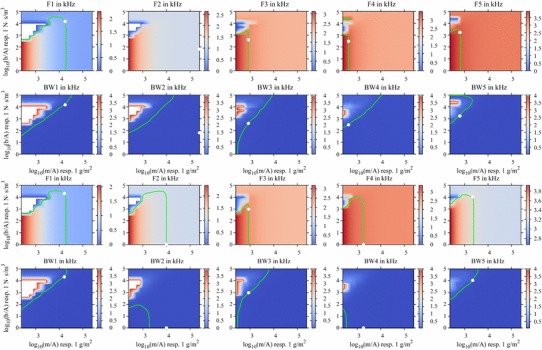
Objective functions for formant frequencies F1–F5 (*first* and *third row* of diagrams) and bandwidths BW1–BW5 (*second* and *fourth row* of diagrams) in case of the vowel /a/. Data are shown for speech mode in the *first* and *second row* and for the singing mode in the *third* and *fourth row* of diagrams. Here, the dependency of each formant/bandwidth on the dissipation ($b/A$) and inertia ($m/A$) at the VT wall is shown. The *green lines* in each diagram indicate the value as determined with DECAP, which is assumed to be the optimal value. The *white-filled circles* in each diagram mark the intersection of the matching pair of *green lines* and is identical to the optimal combination of $b/A$ and $m/A$, where both the formant frequency and bandwidth are in agreement with the DECAP values. In case of no intersection, we defined the nearest neighbours of each pair of *green lines* to be the optimal solution. In case of a missing *green line*, where no solution with the wall impedance approach used in the model is found, the greatest value of $m/A$ is taken into account. *White* islands in the *plots* indicate non-meaningful values in case of high damping $b/A$ and low inertia $m/A$, where the characteristics of TF is completely changed and no direct mapping of the peaks is possible. (Remark: the results of vowel /i/ and //, analogous to the vowel /a/, are available from the authors on request)

In order to fit the model to the desired values of the formant frequencies and bandwidths as determined with DECAP, the parameters $b/A$ and $m/A$ were varied for all four models of the subject’s VT. Therefore, we obtained multiple objective functions for the five formants, five bandwidths, two vowels and two modes of voice production. A representative selection of these functions is shown in Fig. 5 for vowel /a/. It is obvious that for almost all formants and bandwidths, an adequate set of parameters was found, indicated by the intersection of the corresponding optimal values as shown with green lines in Fig. 5. Additionally, these intersections correspond to local minima of the objective functions. In some cases, for example, for the second formant of the spoken /a/, no intersection and, therefore, no optimal parameters were found. Here, the value of F2 determined with DECAP is smaller than the minimal value which can be generated by the model in case of acoustically hard VT walls.

**Fig. 6:**
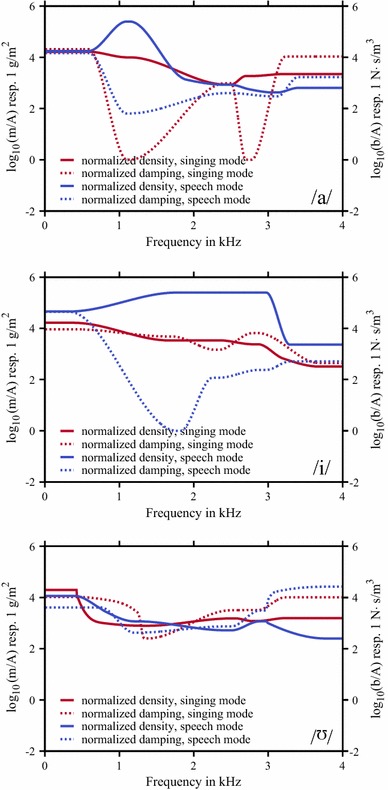
Frequency-dependent functions of $m/A$ (*solid lines*) and $b/A$ (*dashed lines*) for all models as denoted in the legends. The optimal values as determined in Fig. 5 are connected with smooth spline interpolation. To avoid instabilities caused by great derivatives of the functions in the vicinity of the optimal values (width of the bandwidth), the functions are held constant. To avoid non-plausible extra peaks in case of great formants shifts, the spline functions were slightly adjusted between the formants. In singing mode for frequencies greater than 0.5 kHz, $m/A$ is nearly constant but differs between vowels. In general, there is no clear conformity for both, the different vowels and modes

**Fig. 7:**
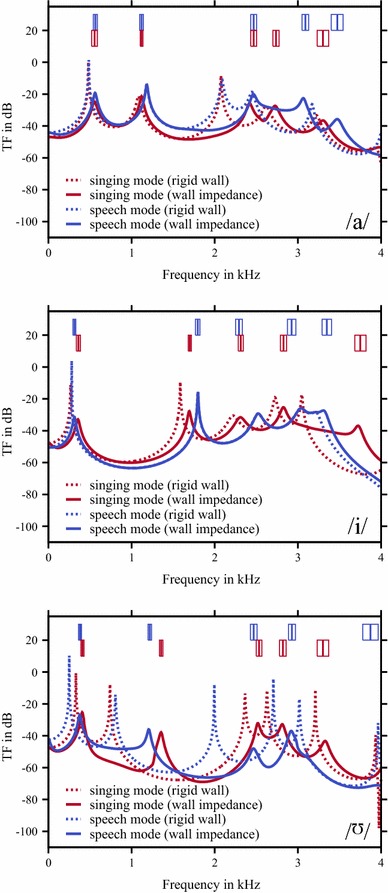
Enhancing of the results of all models by applying optimised mechanical impedance properties of the VT wall (*solid lines*) in comparison with acoustically hard VT walls (*dashed lines*). Analogue to Fig. 4c, the formant frequencies and bandwidth determined with DECAP are shown with boxes (*blue boxes* and *line colours* corresponding to spoken vowels and *red colour* to sung vowels). In general, the peaks of the TFs are shifted towards the position as determined with DECAP and no additional and, therefore, un-physiological formants are generated. In case of vowel // (speech mode, rigid wall condition), F5 was suppressed by introducing the mechanical impedance.

**Fig. 8:**
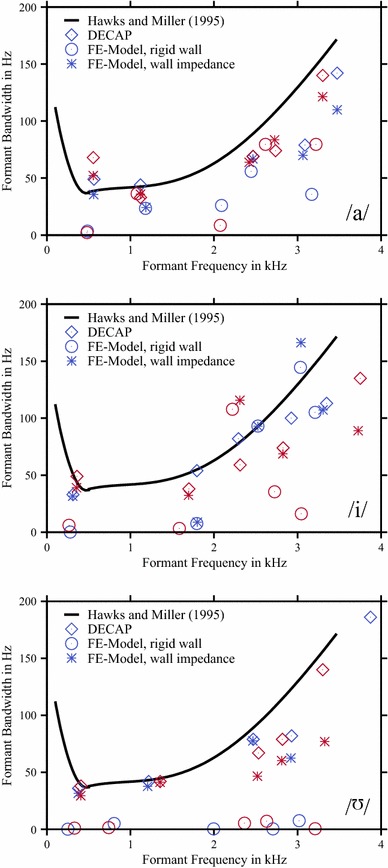
Formant bandwidth as function of formant frequencies for the vowels /a/ and /i/ as denoted in each diagram. *Dark blue* symbols correspond to spoken vowels and *red symbols* to sung vowels, respectively. As shown with *circles*, there is partly a large discrepancy to the values determined by the inverse filtering method (DECAP, denoted with *diamonds*) for the model values assuming acoustically hard VT walls (denoted with *circles*). In contrast, as denoted by *stars*, the models can be significantly improved by introducing the complex impedance of the VT wall. For comparison, the fitting functions given by Hawks and Miller (1995) are shown with *black solid lines*. For the vowel //, the bandwidths in case of rigid walls are all very small. Due to the nearly closed lips, the damping of outgoing waves is not sufficient to generate more realistic bandwidths

**Fig. 9:**
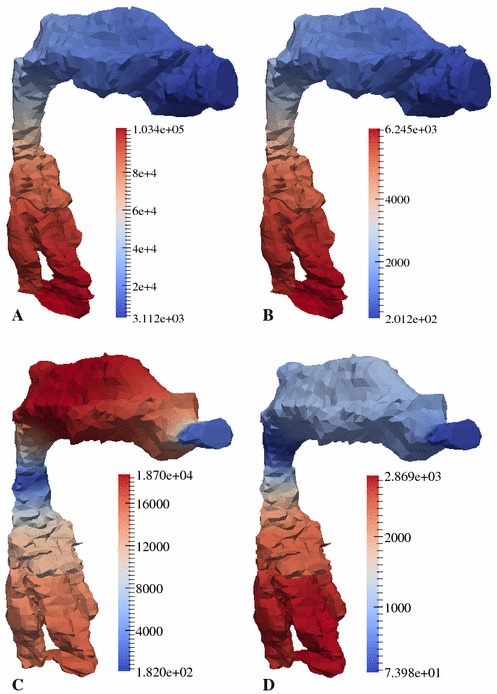
Pressure distribution (amplitude at 1 m/s acoustic source flow $\tilde{f}_0$) for F1 of the sung vowel /a/ (**a, b**) and F2 of the sung vowel // (**c, d**) assuming acoustically hard walls (**a, c**) and an impedance model at the VT wall (**b, d**). *Red-coloured* areas denote greater pressure than *blue* ones. Whereas in case of a slight shift of the formant (see Fig. 7), the absolute values of the pressures are changed (**a, b**)—dependent on the chosen wall conditions—the topology of the whole pressure field is only slightly affected. That indicates that in this case, introducing of mechanical impedance properties of the VT wall preserves the basic physical properties of the VT. In case of a large manipulation of the position of the formant, the absolute values of the pressure are also lowered in case of non-rigid walls (**c, d**). But, here, the topology of the pressure field is changed substantially

It turned out that the optimal set of parameters corresponding to the individual models, showed a frequency-dependent behaviour (Fig. 6) where the inertia $m/A$ of the VT wall was increased in the mid-frequency region of about 1.5–2 kHz (vowel /i/, speech mode) or was nearly constant (for example, vowel //, speech and singing mode). But in contrast, for the losses $b/A$, there was no significant tendency in the frequency behaviour observable. Applying these VT impedance functions to the individual models, an enhancement of the calculated TFs in order to match the characteristics as determined with DECAP was observable (Fig. 7), as expected. In Addition to the graphical depiction of the TFs, the model results, considering the complex impedance of the VT wall, in terms of formant frequencies and bandwidths are denoted with stars in Fig. 8. By applying optimal mechanical impedance properties of the VT wall, the mean deviation of formants and bandwidths were significantly reduced from a several hundreds of Hz to only a several tens of Hz (see Table 3). It should be noted that the specific pressure characteristics of each formant were not changed by introducing the wall impedance. For example, the pressure distributions with and without applying of mechanical impedance properties of the VT wall for the first formant of the sung vowel /a/ and the second formant of the vowel // are shown in Fig. 9. According to the modifications of the TFs shown in Fig. 7, the absolute values of pressure dropped to lower values because of additional losses in the VT wall. Comparison of the related distributions shows that the application of the non-rigid wall condition can change the overall pressure characteristics slightly compared to acoustically hard walls. That means, the formants were not only shifted in the frequency domain. Further, no generation of additional formants occurred.

## Discussion

### Data acquisition

To obtain the realistic geometry of the individual VT for different vowels and mode of voice production, the MRI procedure was designed to analyse the spatial size and arrangement of the laryngeal and the pharyngeal regions with a resolution of about 1.3 mm. To segment the correct inner boundaries, we used a semi-automatic approach (Poznyakovskiy et al. 2011) which is based on the empirical definition of a central pathway along the VT in the sagittal plane and the shape of a cross-sectional area of the VT. The accuracy of the automatic segmentation of the whole tract was controlled manually in all cases. Special attention was given to the lip region, where the automatic procedure fails. Here, the wedge shape of the mouth opening was added manually to the model. It should be noted that this mixture of manual and semi-automatic approaches could lead to small deviations regarding the correct geometry. Further, it should be noted that three-dimensional time-resolved MRI scans are restricted to a few frames per seconds. Therefore, the glottal region with oscillation frequencies greater than 100 Hz is always blurred. For this reason, we made an exhaustive verification of the segmented surfaces by visually inspecting the segmentation results with the pictorial data provided by the MRI.

To avoid unexpected artefacts, we did not smooth the surfaces before exporting to the finite element solver. Considering a resolution of about 1.3 mm and the speed of sound of 350 m/s, the critical frequency for resolving these uncertainties might be much higher than the frequency range we analysed. More problematical are uncertainties caused by periodic and/or transient changes of the VT geometry. A source of periodic changes, especially in the supra-glottal zone, could be muscle activity which results in the vibrato (in the singing mode exclusively, see Arroabarren and Carlosena (2007) for an overview). These periodic changes result in amplitudes of a few millimetres with a frequency of about 5–7 Hz. But by assuming maximal changes of about 2 mm, these variation might have an effect at frequencies much higher than 10 kHz, which means that the acoustic properties of the VT in the frequency range lower than 4 kHz are unaffected by the vibrato.

Further, the determination of the correct size and shape of the cross-sectional area of the glottis is problematical. Within one time slot of MRI recording, the vocal folds open and close about two thousand times so that we cannot detect the correct geometry and only a blurred snapshot representing the averaged position of each vocal fold over the time can be obtained. The significance of this assertion will be discussed in the next subsection.

Transient effects might happen because of the long acquisition time of up to 9.2 s during MRI recordings. For this reason, we decided to choose a subject with a trained voice, where geometrical changes of the VT morphometry are less likely to occur. In sum, the quality of the morphometrical measures seemed adequate for our approach.

For a trained voice, it can be assumed that vowel production and voice modes (and therefore formants and bandwidth) are only slightly affected by the different measurement environments. Moreover, the vowel characteristics we extracted from the acoustical data should strongly cohere to the pictorial data we got from the MRI.

### Finite element modelling

One critical point in using numerical procedures such as the FEM is providing a mesh where the size of the elements is small enough to resolve the pressure field as accurate as necessary and, concurrently, minimise the calculation time. In our analyses, the maximum frequency was 4 kHz. Considering a sound of speed $c$ of 350 m/s, we obtained a minimal wavelength of 8 cm, which is somewhat greater than the maximal element size of about 1 cm. Therefore, we obtained at least eight elements per wavelength, which indicates the capability to solve the physical problem sufficiently. The degree of freedom of our models is in the order of 30,000, independent of the vowel and mode.

To calculate the VT TF, the only source we took into account is a constant flow at the glottis. We neglected any additional source which might result from local turbulences of the airflow close to the glottis (Mattheus and Brücker 2011). These additional sources may have an effect at higher frequencies around 2–4 kHz and may influence the breathiness of a vowel-like phonation.

We are aware of the fact that the boundary condition we choose at the glottis is not able to capture the entire mechanism of sound generation within the human glottis with all its details. Here, the pressure difference between the lungs and the surrounding air drives the fluid flow through the glottis. This airflow excites oscillations of the vocal folds. These oscillations affect the airflow again, so that a pulsating fluid flow through the glottis results. This transient flow regime causes pressure fluctuations and variations that partly radiate as sound waves. It is important to point out that hydrodynamic and acoustic variables differ by orders of magnitude for a low Mach number that applies for the glottal flow during phonation. Therefore, hydrodynamic pressure and velocity values do not equal the acoustic pressure and acoustic flow. Despite the knowledge of these complex fluid dynamics inside the VT, according to Zhao et al. (2002), we decided to approximate the effective acoustic source by an acoustic monopole which is equivalent to an acoustic flow at the glottis.

The sound transferred by the VT depends on the individual impedances acting on the interface to the surrounding environment (Motoki 2002) and the coupling to the body along the VT (Sondhi 1974). Coupling to the surrounding air around the head depends on the shape and size of mouth opening and the frequency range observed. The results of our study confirm the assumption that the sound source is in good agreement with the assumption of a monopole at the lips. As shown in Fig. 4, example shown for one specific geometry, there is no big difference between the VT TF in case of a rigid piston acting into an infinite baffle, its low-frequency approximation, and the boundary conditions for forcing the absorption of spherical waves, respectively. Obviously, the ratio of the real to the imaginary part of the different impedance approximations was similar in the frequency range we observed. It should be noted that the direct comparison between these approaches has some limitations due to the restriction to a specific type of outgoing waves. To address this problem, an additional volume around the VT/Head that includes infinite element approaches (see Retka and Marburg (2013) for an overview) or perfectly matched layer approaches (Arnela et al. 2013) is needed. It can be concluded that in the investigated frequency range up to 4 kHz, not the shape of the lips controls the impedance but mostly the size or the area of the mouth opening Arnela et al. 2013.

**Table 3:** Comparison of the mean deviation from the DECAP values in the F–BW space of all formants for both vowels and voice modes before and after enhancing the model by introducing a mechanical impedance model to the wall with optimal parameters

Vowel	Singing mode		Speech mode	
	Hard (Hz)	Include mechanical impedance (Hz)	Hard (Hz)	Include mechanical impedance (Hz)
/a/	152.1	20.2	300.1	28.7
/i/	230.9	26.8	116.5	91.4
//	250.4	24.6	427.6	33.3

As shown in Sect. 3, the formants and bandwidths cannot be modelled correctly without considering the impedance of the VT wall. Because of the complexity of the surrounding structures containing a great number of different muscles and cartilages and various scenarios of the interaction of these structures during phonation, for simplification, we used a local impedance approach, that means, that the impedances can be considered as an array of mass–damper systems which interact with the VT, but do not interact with each other (Marburg and Anderssohn 2011). By considering of the low shear stiffness of biological tissues, this approach might be a plausible approximation. Furthermore, for simplification, we assume that these impedance values are globally constant, but consequently, as shown in Sect. 3, this results in values which are frequency dependent. Similarly, Sondhi and Schroeter (1987) stated that frequency-dependent elements would be much more appropriate than an ordinary compliance mass resistance system. Additionally, Hanna et al. (2012) could show that introduction of a constant mass–spring–damper system at the wall is a good approximate for the low-frequency behaviour for certain vowels and simplified geometries. Here, in case of acoustically hard walls, one formant is missing (see Sondhi 1974).

The approach used in this study to consider a damped and inertial behaviour of the VT wall leads to values for $m/A$ in the order of magnitude of about $250\,\hbox {g}/\hbox {m}^3$ up to $2.5\times 10^{5}\hbox {g}/\hbox {m}^3$, and values for $b/A$ in the order of magnitude of about 65–$4.5\times 10^5\,\mathrm{{N}}\,\hbox {s}/\hbox {m}^{3}$ (see Fig. 6).

As mentioned before, the impedance at the wall can be considered as an array of local mass–damping systems, acting independently of each other. Assuming low shear stiffness, which is typical for biological tissues, an analogical continuum model can be formulated by introducing an ordinary isotropic viscous element of length $l$, a dynamic viscosity $\eta$ and a density $\rho$. The relationship between the parameters $m/A,\,b/A,\,l,\,\eta$, and $\rho$ is given by $m/A=\rho \cdot l$ and $b/A=\eta /l$.

The VT is completely covered with mucosa (Mescher 2009) with a thickness which is assumed to be in the order of 3 mm (Ueno et al. 2011) and a density of water of $0.001\,\hbox {kg}/\hbox {m}^{3}$. If we consider the masses per unit area, we computed masses to be optimal for all the models in the range from 252 to $251{,}189\,\hbox {g}/\hbox {m}^{3}$; we can calculate effective thickness values $l$ of about 0.25 mm up to 0.25 m. That indicates that the mucosa is affecting the VT TF at frequencies where $m/A$ is lower than $3{,}000\,\hbox {g}/\hbox {m}^{2}$.

Furthermore, the dynamic viscosities $\eta$ are in the order of magnitude of $190\,\hbox {mPa}\,$s–$134\,\hbox {Pa}\,\hbox {s}$, which is (much) higher than the value of (isotonic) water. Further, the values for $b/A$ we found are lower than the real value of wall impedances of about $8\times 10^{4}\,\hbox {N}\,\hbox {s}/\hbox {m}^{3}$ recently used for models of the VT (Švancara and Horáček 2006; Arnela and Guasch 2014).

These estimates show that the values for $m/A$ and $b/A$ calculated in this study are in a confidential order of magnitude. However, it should not obscure the fact that the global approach for the VT impedance is not capable to give inside into the intrinsic fluid–structure interaction acting at the VT wall. Here, instead of frequency-dependent impedances, these values are expected to be a function of the specific location inside the VT. That possibly overcomes the fact that our current approach is not capable to capture the formant frequencies and bandwidths for all cases. Unfortunately, (re-)calculation of these more realistic impedance values as a function of the position within the VT is only possible in case of a completely known sound pressure field (Marburg and Hardtke 1999).

In the high frequency region (around F4 & F5), the presented procedure becomes more disputable. Because of the higher modal density, depending on the parameters (and the chosen fitting procedure) identification and attribution becomes more difficult. One critical point could be the influence of small geometrical variations for instance [after tonsillectomy, see Švancara and Horáček (2006)] and of laryngeal cavities (Takemoto et al. 2006) and their effect on the VT TF.

In mechanical models based on MRI data of a male, Dang and Honda (1997) found anti-resonances in the frequency range of about 4–5 kHz which are associated with the piriform sinus. In contrast, the results of numerical models in Takemoto et al. (2006) revealed an additional formant (F4) which results from the existence of these laryngeal cavities. Both studies have been done without considering the interaction with the tissue at the wall of the VT. In the present study, assuming acoustically hard VT walls, at frequencies greater than 4 kHz, we partially found antiresonances followed immediately by a resonance. This appearance is completely suppressed by applying damping boundary conditions where, moreover, a dominant formant without any antiresonances results. Considering maximal energy transfer in this frequency range, i.e. a maximal output, especially in case of professional classical singing under consideration of the so-called singer’s formant cluster in males, these behaviour seems to be plausible and very effective.

In an experiment presented in Fant et al. (1976), Fujimura and Lindqvist (1971), where the VT of male subjects were excited by a sinusoidal pressure using a tube which was sealed at the lips and the glottis held closed, the authors found a local resonance of about 190 Hz and a bandwidth of 95 Hz at the level of the larynx. This resonance was measured externally at the skin via a piezoelectric transducer. By contrast, in our model, a resonance in this frequency range is not observable. At least three possible explanations for this discrepancy are conceivable. Firstly, the local resonance is caused by specific local impedances seen by the VT as argued by Fant et al. (1976), which was (for reasons mentioned above) not incorporated in our model. Secondly, it might be possible that the absolute value of the impedance at the VT wall— in case of this “reverse” excitation—is significantly lowered because of flabby muscles, especially close to the larynx. Thirdly, in our model, the VT impedance does not consider the gradient of mass and damping from the VT wall to the external wall of the neck, respectively, which means that the mechanical behaviour of the surrounding structures far away from the VT cavity is not influencing the TF of the VT.

## Conclusion

In this article, we present a strategy to enhance MRI-based FEM models of the VT in order to match the formant frequencies and bandwidths as determined by using inverse filtering. It is shown that the mean deviation between the FEM models with acoustically hard VT walls and the inverse filtering approach ranges between 116.5 and 427.6 Hz, depending on the vowel. Introduction of mechanical impedance properties of the VT walls reduces the difference of the inverse filtering approach to values of 20.2 up to 91.4 Hz. Further, significant differences in the wall impedances between the articulated vowel and the voice production mode (spoken vs. sung) are detected. These results indicate that the interaction between the acoustics of the air-filled VT and its surrounding structures is in a non-negligible order of magnitude and contributes to the fine tuning of articulation.
